# From causes to solutions - insights from lay knowledge about health inequalities

**DOI:** 10.1186/1471-2458-11-67

**Published:** 2011-01-31

**Authors:** Christine Putland, Fran E Baum, Anna M Ziersch

**Affiliations:** 1 Southgate Institute for Health, Society and Equity, Flinders University, Bedford Park, South Australia

## Abstract

**Background:**

This paper reports on a qualitative study of lay knowledge about health inequalities and solutions to address them. Social determinants of health are responsible for a large proportion of health inequalities (unequal levels of health status) and inequities (unfair access to health services and resources) within and between countries. Despite an expanding evidence base supporting action on social determinants, understanding of the impact of these determinants is not widespread and political will appears to be lacking. A small but growing body of research has explored how ordinary people theorise health inequalities and the implications for taking action. The findings are variable, however, in terms of an emphasis on structure versus individual agency and the relationship between being 'at risk' and acceptance of social/structural explanations.

**Methods:**

This paper draws on findings from a qualitative study conducted in Adelaide, South Australia, to examine these questions. The study was an integral part of mixed-methods research on the links between urban location, social capital and health. It comprised 80 in-depth interviews with residents in four locations with contrasting socio-economic status. The respondents were asked about the cause of inequalities and actions that could be taken by governments to address them.

**Results:**

Although generally willing to discuss health inequalities, many study participants tended to explain the latter in terms of individual behaviours and attitudes rather than social/structural conditions. Moreover, those who identified social/structural causes tended to emphasise individualized factors when describing typical pathways to health outcomes. This pattern appeared largely independent of participants' own experience of advantage or disadvantage, and was reinforced in discussion of strategies to address health inequalities.

**Conclusions:**

Despite the explicit emphasis on social/structural issues expressed in the study focus and framing of the research questions, participants did not display a high level of knowledge about the nature and causes of place-based health inequalities. By extending the scope of lay theorizing to include a focus on solutions, this study offers additional insights for public health. Specifically it suggests that a popular constituency for action on the social determinants of health is unlikely to eventuate from the current popular understandings of possible policy levers.

## Background

This paper is concerned with lay understandings of place-based health inequalities. In the 21st century there has been greater acknowledgement of the complex interaction of individual (for example diet, lifestyle choices) and social/structural (poverty, service provision) factors in determining patterns of health and illness. Growing recognition of the urgent need for effective strategies to address inequalities in health status and the resulting inequities in access to services and resources has been underscored by the report of the WHO Commission on the Social Determinants of Health which reinforced the importance of expanding our knowledge base and raising public awareness [1]. These concerns have led to a greater focus on health inequalities linked to areas and places. Although it is well-known that people living in more deprived areas experience poorer health, the underlying causative processes in this relationship are less clear [2,3]. Is it the characteristics of individuals living in these areas ('compositional' factors) or characteristics of the areas themselves ('contextual' factors) which are more influential? Research has highlighted important questions about areas and places as social spaces within which people live and interact with each other and with their environment [4,5]. The demand for an understanding of how people's identities, attitudes, behaviours and relationships are shaped by, and in turn shape, the places in which they live, intersects with the literature concerned with the contribution of lay knowledge in public health [6].

Recent decades have seen the development of a substantial body of research concerned with accounts of health and illness from the perspective of ordinary people in Western countries. Within this literature a gradual shift in focus is evident, from the subjective experience of the 'sick role' [7] to studies of how people understand the broader social determinants of health [8-13,3]. This work has shown an increasing tendency for people to conceive of health in multidimensional terms beyond a narrow bio-medical model [14,15], underscoring the relevance of lay ideas for a more rounded understanding of health inequalities. Alongside this development the simplistic presumption of lay and professional/expert views as necessarily polarized has given way to the accumulation of a 'richer, thicker description of lay concepts of health and illness'[16]. The language in the field has expanded from the passive descriptor of 'lay beliefs' to the more compelling 'lay expertise' and 'lay knowledge' [16].

This shift goes beyond mere semantics to conceive a different, more 'nuanced and sophisticated' role for lay knowledge [17]. As such it has the potential to offer insights that complement, challenge or hold a mirror to technical expertise concerning health inequalities. By many accounts, however, few studies engage this potential directly. More than a decade ago, Blaxter [4] observed that with the exception of Calnan's [18] small-scale exploratory study, little attention had been paid to the question: 'How do people themselves think about inequalities in health?' According to Davidson et al. [19] this continues to be the case, with most public health studies approaching the subject obliquely.

Nevertheless, there is a small but significant body of work asking direct questions about how people make sense of the patterns of social inequalities that have been linked to health outcomes [4,6,16,20-22,19]. In this work researchers have a special interest in whether people accept social/structural explanations for inequalities and whether certain groups of people, in particular those with personal experience of disadvantage, are more or less likely to do so. These questions are important because they have implications for the direction of public health policies and programs designed to reduce health inequities. Overall it has been found that people's theories about causes are multi-factorial, although in terms of structural versus individual explanations the results are decidedly uneven. The reference point for much of this discussion is Blaxter's [4] conclusion that lay people, especially those most at risk, tend to display conceptual difficulty with the notion of structural causes of health and ill-health. She observes that the interest in lay views arose principally from the concern that poorer or less educated people are more resistant than other groups to education about 'healthy behaviour'; yet paradoxically their responses tend to echo rather than challenge the powerful trends in epidemiology and health promotion towards 'individualised risk factors' [4]. Hence debates have centred on the extent to which people's responses reflect their own experience of advantage/disadvantage associated with living in 'deprived' areas.

Subsequent studies based variously on quantitative, qualitative and mixed-method designs have produced varying results in relation to these questions. Supporting Blaxter's findings, for example, in multivariate analysis of a postal survey comparing more- and less-advantaged areas in Scotland, Macintyre et al [21] found that 'those more at risk of ill health may be less likely to acknowledge the social gradient in health'. By comparison, using a mix of qualitative and quantitative data from 2 socio-economically contrasting areas within each of 2 cities in the North West of England, Popay et al [16,20] found that responses differed depending on the methods employed. Survey respondents had no difficulty in offering explanations for inequalities, and those living in disadvantaged areas gave prominence to structural factors and aspects of place [16]. In the in-depth interviews, however, resistance to the abstract notion of inequalities in health between areas and social groups was observed [16]. More recently, Davidson et al [22,19] analysed data from 14 focus group discussions with pre-existing groups of people to explore their experiences and understandings of social and health inequalities and the differences between more or less affluent areas. The researchers encountered no reluctance to talk about inequalities amongst the less advantaged, but instead a readiness to discuss the adverse effects on health and wellbeing of structural and contextual features. Moreover, people's own experience of advantage or disadvantage, whether direct or indirect, was found to temper their views. In other words, the people who most readily accepted statements about the influence of area level features on health inequalities were those who lived in more disadvantaged areas, 'together with those from more-advantaged areas with past personal or professional experience of deprivation' [19].

It is noted that comparisons demand careful consideration since the varied research designs and methods used have been shown to influence the study findings [14,21,16,20,22,19]. For example, a self-completion survey may offer greater anonymity and distance, fostering acceptance of structural inequalities amongst respondents. By comparison, the more personal interaction within interviews is thought to evoke a 'moral dilemma' wherein participants' identities become implicated in the experience of disadvantage [16,20,4,23,11,19].

This paper examines these themes in relation to a study undertaken in Adelaide, South Australia. The findings from this research enable discussion of lay knowledge about health inequalities in the Australian context and against a backdrop of policy developments in the public health field. The decade or more following Blaxter's [4] observations coincides with a more pronounced focus internationally on the social determinants of health. This situation invites consideration of whether such increased attention may result in shifts in public understanding. While it is neither possible to draw such conclusions on the basis of a small study alone nor to address it comprehensively within the scope of this paper, the findings presented here are nevertheless powerfully evocative.

Details of the study design are described, highlighting the form of questioning used to elicit participants' ideas about suggested solutions as well as explanations for health inequalities. Findings are summarized in light of issues raised in the literature in respect to the balance between agency and structure and the influence of living in a relatively deprived area on these responses. In addition, the paper examines the relationship between the ways in which people think about the problem of health inequalities and the kinds of solutions that they envisage.

## Methods

The data presented in this paper are drawn from the qualitative component of a multi-staged, mixed-methods research project conducted between 2003 and 2006 which examined the connections between urban location, social capital and health [24]. The data are based exclusively on eighty in-depth interviews with residents in four case study post code areas situated within socio-economically (SES) contrasting (as identified by the Australian Bureau of Statistics Socio-economic Indexes for Areas (SEIFA) - Index of Relative Disadvantage) Local Government Areas (LGAs) in Adelaide. The postcode areas are named after the LGAs in which they are situated: Burnside (high SES), Prospect (moderately high SES), Onkaparinga (moderately low SES) and Playford (low SES). Ethics approval was obtained from the Flinders University Social and Behavioural Ethics Committee.

Recruitment for the in-depth interviews was by means of an invitation extended to postal survey respondents in an earlier stage of the study, who were asked to register their willingness to participate in qualitative interviews. From those who agreed, a sub-sample of 60 (15 in each post code area) was purposefully selected. The SES of the respective post code areas was the central unit of analysis and particular attention was paid to achieving a gender and age balance within each. Given that people on low incomes are less likely to respond to mail surveys an additional 20 interviews (five in each area) were undertaken with people with very low SES (weekly income less than A$250). These participants were recruited through charities, advertising in local newspapers and letterboxing. While the focus of the analysis was on area of residence rather than individual socioeconomic status, it is worth noting that even within the better off areas the study samples for each area included individuals with very low incomes.

The interviews were designed to gather rich, detailed information about participants' experiences and perceptions of their neighbourhood, social and civic life, and the impact of these on their health. They ranged between 1-2 hours in duration and were conducted in the participants' own homes. With the consent of the participants the interviews were recorded using digital audio recorders, and transcribed verbatim. Interview data were analysed with the assistance of Nudist software using a broad 'Framework analysis' [25]. Commencing with a data familiarisation phase, interview transcripts were downloaded into Nvivo 7 and coded into 'nodes' organized according to themes. A 'thematic framework' was developed based initially on the pre-identified 'a priori' topics that guided the interview schedule, mainly corresponding to themes within the literature. As analysis progressed these were expanded to include emergent themes from repeated analysis and refinement of the coding framework.

While data from these interviews have been the subject of reports and publications elsewhere [24,26], responses to a particular line of questioning about 'health and neighbourhood' are the focus in this paper. Following on from questions about perceptions of neighbourhood and participation in social and civic activities, participants were asked to consider what they thought would make their neighbourhood a healthier place to live. A series of questions and prompts concerning health inequalities was embedded in this discussion, drawing on an approach outlined in the literature by Popay et al. [16,20] and later by Davidson et al. [19].

Initially participants were invited to talk about 'why people in some neighbourhoods have worse health than people from other neighbourhoods' using a visual and textual prompt in the form of a newspaper cutting to stimulate discussion. They were shown a newspaper article with the heading 'GREAT DIVIDE: In Mitcham, you'll live five years longer than someone in the Port' and depicting a family of four in front of their home: mother, father and two young children. The caption below the image states that the couple are 'happy with their home, but fear asthma-related symptoms suffered by children...may be caused by pollution'. The article makes reference to a university report on a study commissioned by the State government into inequality, highlighting the widening 'health and wealth gap' in South Australia. The newspaper article was intended as a catalyst for thinking about the causes of inequalities. Participants were encouraged to talk about possible reasons for the kinds of differences cited in the report and to apply these reasons critically to neighbourhood differences more generally. Following some discussion, participants were then asked to consider 'if they were in government, what they would do about health inequalities'. In this way participants progressed from identifying causes to thinking about the kinds of public policy strategies that might be employed to address these inequalities and to engage more fully in the process of theorizing about inequalities. Just as the framing of policy responses to social problems is regarded as a representation of how the original 'problem' is understood [27], so analysis of how lay people construct new knowledge offer insights into their interpretation and response to existing and observable situations.

The two areas referred to in the article, Mitcham and Port Adelaide, are older well-established suburbs and researchers were justifiably confident that all of the participants would be familiar with them by reputation at least. Mitcham is known as a relatively wealthy suburb, with a mix of older style and more modern housing, heavily wooded and situated in the foothills south east of Adelaide city centre. Port Adelaide is an older western suburb known for its heavy industrial development, dockyards and housing for families of blue collar workers, with more recently developed pockets of gentrification. Although many participants did not appear to read further than the headlines in the newspaper article, it should be noted that the text makes reference to a range of factors mentioned in the full State Government report including pollution, income, education, life expectancy, smoking, transport, employment, housing, crime, gambling, child abuse, diet, obesity and access to services. The prompts by the interviewer aimed to engage participants in discussion beyond the specific details of Mitcham and Port Adelaide, and their own area of residence. Nevertheless in many cases they did spontaneously relate the issues to their own area and to personal experience. Findings are summarised below with selected quotes to illustrate the spread of responses as well as to highlight particular cases. Participants are identified by pseudonyms, age and post code area of residence (B = Burnside, O = Onkaparinga, P = Playford, Pr = Prospect).

## Results

The data presented in this paper are drawn from interviews undertaken with the total number of 80 participants, distributed as follows: Burnside (10 females; 10 males), Prospect (11 females; 8 males), Playford (11 females; 9 males), Onkaparinga (11 females; 10 males). Participants in this study were mostly willing to accept the premise of the newspaper report about the differences between Mitcham and Port Adelaide, despite some initial hesitancy:

I don't even know if that's true; like how do they even come up with things like that? (Kirsty, 28, P)

...how can we compare, because you only get one life and when that's gone you can't say: 'well I'll come back and live in this [other] one now and see if I can live another 10 years longer'. (Ralph, 56, Pr)

Once engaged, the overall scope of explanations canvassed was broad, ranging from factors related to the physical, ecological and social environment, described here as social/structural, to those favouring individualized causes. Our principal interest in this paper is in the balance between these categories, and the extent to which views depended on whether they lived in a more or less deprived area. Caution should be exercised, however, in such a qualitative study encouraging reflection and participants' ability to revise their responses in the course of the questioning. Initially, therefore, we will define our reference to 'social/structural' and 'individualised' in terms of examples from the study participants, and then proceed to examine the balance between the two in their responses.

### Individual and socio-structural explanations for health inequalities

In this study social/structural explanations were identified as those based on an understanding that patterns of inequalities shape behaviours and limit the control of individuals over their lives. Table 1 shows the wide range of responses that were categorised as social/structural.

**Table 1:** Social/structural explanations for health inequalities based on characteristics of area and populations

*Poor quality of the ecological environment in Port Adelaide*	...there is a lot of industry down there that's belching stuff out into the atmosphere and pouring it into the river and I mean that can't be good for you. (Justin, 50, B)
*Comparative amenity or visual quality of trees, parks and buildings*	... you know the eastern suburbs are pretty green and offer a lot more healthy environment than somewhere that is heavily industrialised. (Maxine, 61, Pr)

*Comparative service provision*	It's a lot harder to get transport in those areas, public transport isn't as good as it might be for example... (Jason, 58, B)

*Unemployment*	Unemployment rates could have an impact, especially if you're unemployed and your future prospects don't look that great and the choices you make within that context. (Rebecca, 35, O)

*Financial insecurity*	I think worry must play a big part in general health. If you have a continual concern about...whether you've got enough money to see you out to the end of the week. (Drew, 78, B)

*Level of income and access to health care*	In Mitcham everybody has got the money to afford the appropriate healthcare... so they can afford to look after themselves, whereas in Port Adelaide you have got people on the social welfare, you can't get your dental fixed for 5 years because they are on the free list. (Evan, 51, Pr)

*Social environment - crime*	...living in a high crime rate or um people are being threatened on the streets, then they're in a lot worse position than if they're living in a stable community. (Michelle, 33, Pr)

*Social environment - neighbourhood supports*	... if you can talk and get on with your neighbours, that's a big thing to make you happy in your own environment and therefore you live longer. (Catherine, 68, Pl)

By comparison with social/structural explanations, the range of types of responses based on individual characteristics and behaviours as the direct cause of health inequalities was narrower in this study as shown in Table 2.

**Table 2:** Individual explanations for health inequalities based on qualities of individual residents

*Genetic characteristics*	.... I think it really depends on your genes. (Cathy, 25, Pl)
*Knowledge about healthy eating*	Well probably diet.... I would have to say it's a lot to do with the food that people eat, it's ignorance. (Amanda, 60, Pl)

*Family values*	...the family that you grow up in...they have certain values... knowing that if you want to be healthy you need to eat right and play sport...if you grow up in a family environment like that then you just think that's normal; that's what you continue to do and that becomes generational. (Rhonda, 26, B)

*Lifestyle choices*	Their [Port Adelaide residents'] priorities tend to be more 'go to the pub and play darts with your mates and have a barbecue of greasy food and a few beers'... I think if you can buy beer and cigarettes and greasy chops you can buy healthy food. (Nathan, 59, B)

Within the range of responses listed in Tables 1 and 2 some explanations were more prominent than others. Among the social/structural explanations, for instance, comments about industrial pollution in Port Adelaide and issues associated with income levels were most prevalent, echoing the emphasis in the newspaper article. By comparison, mention of the social environment and service provision was less pronounced. Within the 'individual' explanations there was little mention of genetic characteristics while diet and lifestyle choices were repeatedly cited as determining factors.

The fairly even distribution of responses *between *the categories is interesting insofar as it relates to the question of participants' willingness to talk about social/structural factors. Responses were evenly divided into three main groups: those who stressed exclusively individual explanations, often disregarding or dismissing social/structural factors; those who identified predominantly social/structural factors; and those referring to both individual and social/structural factors. There were no discernible differences according to demographic characteristics like age and gender in the distribution of explanations among these groups.

Two points of interest arise from this pattern. Firstly, bearing in mind concerns raised in the literature about the influence of research design on findings, several features of this research may be regarded as 'priming' the participants to offer explanations based on social/structural factors. These include the overt focus on 'area' in the overall study, the orientation of interview questions towards social and physical features of places, and the newspaper article itself concerned with the comparative quality of suburban environments. Accordingly, when asked to talk about reasons for the reported differences between the areas, the tendency to cite aspects in the physical environment such as pollution, or related social effects of poverty, unemployment and poor housing, were to be expected. The more surprising response in this context was the equally strong tendency to emphasise exclusively individualized explanations, or to gravitate away from social/structural towards individualized causes in attempts to pinpoint connections with health outcomes.

This last point draws attention to a second feature in the data: the inclination for participants to weave a multi-dimensional narrative as they strived to make links between their observations and understanding of complex causes and effects. Melanie's detailed account illustrates how this emerged:

In a poorer suburb they tend to spend their money on different things that would be perhaps more important to them. If they're in a financially poor situation I think they need pleasures, so smoking, drinking, gambling. I'm not saying this is bad, but they're the things that would give them pleasure. Sex and food is probably down lower than that. There are far more problems and worries for them if they've got monetary problems and they often run several cars, so that paying Peter to pay Paul to pay...

I think that they seem to be in turmoil of living. I think happiness comes into it. There would be some people in those areas that would be quite content and quite happy...even if you're poor I consider you can be in good health and live a happy life. But so often I suppose...they're living in smaller houses, perhaps more people in the houses, children that are having problems, probably their intelligence levels could be lower, it may not always be, but I do think low socio-economic groups tend to and I believe that there will always be 20% who are going to need help and I don't think you can help that. ... (Melanie, 72, B)

Typically, participants such as Melanie canvass a range of social/structural and individual factors, picking up on the various triggers cited in the newspaper article. However, like others who felt confident that the different socio-economic profiles of Port Adelaide and Mitcham were linked to living conditions and therefore to health behaviours and outcomes, the precise mechanics of these connections remained elusive. Thus in the end many settled for a sense of the inevitability of inequalities. Participants who drew on both categories in their explanations would often begin by citing social/structural factors as the root cause, then lean towards individual choices and behaviours to illustrate how these factors led to poor health outcomes. Marlene provides a clear example of this shift:

A lot of that is just lack of opportunity, seeing themselves not having opportunity, that sense of hopelessness and helplessness....they just don't eat properly, they don't exercise and that's physical but it also compounds on their mental health. (Marlene, 54, O)

### Influence of relative disadvantage on responses

Another central question in the literature is whether people from more deprived areas are more or less likely to be open to social/structural explanations for health or ill-health. There are two possible ways to discern such an influence in this qualitative study: by participants' current area of residence; and, from the references they make to personal experience in their responses.

Regarding the first, the distribution of social/structural versus individual explanations was broadly similar across the four post code areas, and the data did not offer any distinct insights. People in the lowest SES post code area of Playford were slightly more inclined to challenge the validity of the claims in the newspaper report than in the other areas, while in Burnside, the highest SES post code area, acceptance of the claims was almost universal. Overall, however, the differences were not pronounced.

In regard to the second influence of personal or family experience there were greater differences, however. Participants from the lower SES post code areas of Playford and Onkaparinga appeared to draw more readily on such experiences to illustrate their theories about reasons for inequalities regardless of whether they favoured individual or social/structural explanations. Some, like Scott, referred to people they knew who were struggling financially:

I can talk about some of the families I know of. Once they are just starting to get their head above water and they're getting the bills out of the way, all of a sudden another pile of bills comes in and it does take its toll on your life. (Scott, 50, Pl)

Others like Ruth reflected on their own situation:

We've been in the position where we've had to be on the dole before, been out of work for a year...and we still managed to...keep a roof over our head...with 3 teenagers under the roof! (Ruth, 42, O)

By comparison, participants from the highest SES post code area of Burnside rarely referred to personal or family experiences or to (positive or negative) features of their own area in order to support their arguments about causes, except to draw on their professional experience of having worked in a relatively deprived area. Linked to this finding two dominant themes emerged. The first was a tendency to stress 'what is wrong with Port Adelaide' compared to 'what is going right in Mitcham'. This pattern was pronounced, with most participants talking exclusively about the problem with 'poor people' and 'poor areas' rather than the possible impact of factors that may be seen to promote good health. Hence, living in a high SES area such as Burnside was not recognised as a source of anecdotes or illustrations relevant to 'the question'. Once again, this emphasis is consistent with the 'deficits' orientation of the newspaper article.

The second theme was the use of language evoking a 'them' and 'us' orientation. Sophia, for example, attributes poor health to poor choices resulting from a lack of knowledge about what constitutes healthy behaviour:

That is what I hear, I don't know...in Port Adelaide...they don't earn as much. They like to gamble and drink...having enough money, you can afford to buy better things or be educated to eat better things.... There are people who have got no idea, if you talk sometimes to people, what is healthy and what is not. They eat chocolate, they eat lollies. I mean I love chocolate too, don't get me wrong. (Sophia, 57, Pr)

Sophia acknowledges socio-economic factors, and rather than blaming poor attitude or irresponsibility she points to 'ignorance' to explain 'unwise' lifestyle choices. She is sympathetic to the predicament of hypothetical 'poor people', and to avoid appearing judgemental she shares her own personal 'weaknesses'. Nevertheless, she is careful to portray people living in 'poor' areas as fundamentally different from herself (that is, 'them'). The latter was a familiar refrain among participants from all areas, including Playford:

They want money, they haven't got money...but they're just living off the dole and not helping themselves...I think they're taking the will away from the kids to work. They've given them too much. (Jack, 66, Pl)

The subtext in such accounts reads: 'if I take responsibility for myself then this will not happen to me'. As a means of placing moral distance between oneself and 'poor people and places', it illustrates the moral dilemmas that attend discussions of health inequalities.

### Suggestions to address inequalities

Following discussion of the explanations for health inequalities, participants were asked to consider what they might do to address the differences between Port Adelaide and Mitcham if they were in government. In many cases participants had already ventured to suggest strategies to address the problems previously and the two questions merged. Initially, there was some pessimism about the ability of governments to solve the problem:

Nothing because there's not much we can do about how long people live. (Kelly, 22, Pl)

...you can't just go in and say 'right, well we'll give you all good housing'...it's a bigger thing than that isn't it. (Melanie, 72, B)

They're very complex answers because you've got to go through a lot to make one change. (Evette, 34, O)

Aside from such misgivings, participants attempted to offer thoughts about how government could act to address health inequalities. The extensive range of explanations that had been offered by participants contracted to a narrower selection of 'solutions' as shown in Tables 3 and 4.

**Table 3:** Solutions to health inequalities based on social/structural explanations

*Physical environment*	...I immediately think of the Port and the industry that was there and the contaminants in the soil... that would come down to building regulations. (Tim, 34, Pr)
*Social networks*	I'd be looking at building up the social capital. Developing networks of people and communities and um, trying to improve people's participation in community and in education. I'd certainly be making public transport free. (Michelle, 53, Pr)

*Social and financial supports*	...more support and more money available to support people who have fallen through the cracks for whatever reason... (Jessica, 50, B)

*Services*	...childcare...transport...and more local doctors... (Evette, 34, O)

*Income levels*	Institute a wealth tax. (Frank, 52, Pl)

**Table 4:** Solutions to health inequalities based on individual explanations

*General Education (schooling)*	...you get them to value education and seeing education as a means to get them out of the rut, getting themselves more options... (Marlene, 54, O)
*Education in life skills*	I'd start teaching children from primary school age about nutrition and how to prepare food...(Beatrice, 46, Pr)

*Family intervention*	I think family intervention with people going into the homes and actually working with families could do a lot...but I really do believe that much more personal interaction in a number of these families would help. (Margaret, 68, B)

*Health promotion campaigns*	I suppose try and initiate some good healthy campaign in the Port Adelaide region...put a program in place to get people out there exercising. (Justine, 26, B)

There was considerable resistance to the idea that governments were responsible for solving the problem for a range of reasons. Jennifer, for instance, thought that the government's role should only go so far and that the critical factor was having a 'positive attitude' and '*helping oneself'*. She draws on her own experience to stress the importance of 'strength of character':

I don't know how we did it but... you just sort of pull your head in and you do manage and we still managed to stay healthy... I could have just played the victim and said "Oh poor me I can't do this anymore"... but you know you just do it. So it still is an attitude thing. (Jennifer, 40, O)

Others, mainly few older males, envisaged a very different kind of role for government, recommending draconian measures directed at changing the behaviours and attitudes of the kinds of individuals whom they believed were responsible:

I've certainly got ideas. Firstly I'd bring in national conscription [armed forces] again.... I think the government gives them far too much... People have got no idea of responsibility... (Nelson, 73, O)

I wouldn't allow them ['single mothers'] to have babies...it's disgusting and they've broken down the family unit. (Jack, 66, Pl)

The difficulties in articulating the pathways from social/structural causes to health outcomes observed above were compounded as participants attempted to identify solutions. The overwhelming response was a call for education of individuals - specifically parents and children - about healthy living. It was common for those who had entertained the possibility of complex causes to explain the differences in life expectancy between the areas of Mitcham and Port Adelaide, to become uncertain about how to translate this understanding into a compatible government response. Phil offers one reason for this as he considers education more achievable than attempting to address broader issues:

Well, they want to educate people to be more conscious of the things that they can undertake themselves; give them better choice of food and recreation to look after your own health really; that's a good start. Moving the factories and things might be a bit more difficult but things that people choose for themselves, things like smoking. (Phil, 54, Pr)

Others, like Lynn who had attributed the differences in health to a range of factors including high levels of pollution-induced asthma in Port Adelaide, opted for educating children about healthy behaviours:

I think you have to start at school, you have to start training them. I mean I am thinking of these movies in America about the real low life where they have taught the kids the basics and what a difference it made... (Lynn, 59, Pl)

This data suggests that participants appeared to be drawn to singular strategies which may seem more tangible and within the control of ordinary people. Peoples' ability to move backwards and forwards between apparently opposing concepts has been noted [4]. Additionally, in this study there was a strong sense that participants lacked a vocabulary about strategies that might align with their analysis of the problem. Gordon illustrates this struggle to negotiate a balance between individual and social responsibility:

I would be concentrating in those areas and those low income earners. Just basically hear what they're saying. They'll give you the answers. You just need to speak to them. But then you need to manage what they're saying. I think...it's a hard question. It's almost like an impossible task. I don't know. Benefits for a start. With the benefits, I'd be educating them. You've got to compromise. You just can't give it to them. You've got to work it with them - work in with them. If they want to help themselves, they play the game. If they don't want to help themselves, then they've got to put up with how they're living. Offer them education, offer their kids education, try to keep them in school. And offer them a little bit more money. But, they can't breach it. They can't afford to breach it. A lot of people, you give them the opportunity, they'll do it. They'll try anyway. But there's a lot of people that won't...- money is a big issue for these people. (Gordon, 42, O)

Gordon wants to be inclusive but finds himself reverting to the theme of 'them' and 'us': 'they lack knowledge'; 'they don't help themselves'; 'they are bad parents'; 'money is a big issue for them'. Such a shift allows for the construction of an archetypal 'poor person' who is vulnerable to both the social/structural pressures of living in a disadvantaged area, as well as to individual weakness. Thus the moral dimensions of causal explanations identified above became even more prominent in discussion of solutions, as participants adopted moral positions in relation to 'the problem' and therefore towards this 'poor person', who is consistently portrayed as someone markedly different from themselves.

## Discussion

While the literature indicates uneven findings about people's willingness to discuss health inequalities, in this study a majority of participants engaged readily with this subject, citing explanations that reveal some awareness of social/structural issues. Comparison between studies with different research designs, however, has been shown to be unreliable [16,20,22]. In this study the overall focus on location and social capital, the form of questioning about health inequalities, and use of a media report contrasting two areas, might be expected to drive discussions towards social/structural issues. Similarly, questioning by interviewers encouraged reflection on issues in the abstract rather than on personalized accounts, which may also have compounded the distancing effect and resulted in the relatively high level of acceptance of social/structural causes. Other findings are more difficult to account for in terms of research design, however. For example, the strong emphasis on exclusively individual causes is surprising in light of the drivers towards social/structural factors, suggesting the residual 'power of individual agency' described by Popay et al. [16]. Meanwhile Blaxter's [4] observation of people's ability to simultaneously entertain seemingly opposed theories is evoked in the subsequent propensity to drift away from social/structural explanations and towards individualized solutions.

The literature also showed conflicting findings concerning the question of whether relative dis/advantage or experience of living in a deprived area is linked to people's willingness to acknowledge socio-structural factors as determinants of health inequalities [21,16,19]. In our research we were able to discern no appreciable differences between the responses from participants in the different SES post code areas. Participants' inclinations to explain inequalities based on social/structural or individual factors did not appear to be tempered to a great extent by direct experience of disadvantage or indirect witnessing of disadvantage, unlike findings by Davidson and colleagues [19,22].

By the same token it was clear that participants from the lower SES post code areas were more inclined to draw explicitly on examples from their personal or family experiences, as well as on their observations of others living in a relatively deprived area, in order to justify their explanations. This was likely to be linked to the strong overall tendency to focus on the deficiencies in Port Adelaide rather than the strengths in Mitcham; that is, a preoccupation with 'health deficits' as opposed to a positive notion of health and how it may be enhanced. The construction of 'poor people' and 'poor places' in terms of 'them' not 'us' was a similarly compelling theme which pervaded the data uniformly across all post code areas. In light of the profound 'moral connotations' for places and the people living in them that have been associated with an acceptance of health inequalities [4,23,20,11,19], this approach may be seen as a means of achieving a moral distance from the problem, and from personal identification with the perceived deficiencies mentioned above.

Posing a question about how governments should address health inequalities in our study was designed to elicit more developed theories about causes and solutions. It served to illuminate how participants arrived at their understandings. The limited pallet of ideas about appropriate public policy responses that were canvassed by participants stood in stark contrast to the broad range of explanations that had been offered. The drift towards more privatised solutions with their presumption of individual responsibility for the problem was marked, as participants, including those who had initially cited social/structural causes, sought solutions in behaviour change strategies - predominantly education about lifestyle choices for individuals and families. This seemed a less challenging option than the daunting task of changing structural factors, as other studies have hinted [8].

Several limitations in the study should be noted in relation to the above discussion. As a qualitative study, it has been possible to make comparisons with other similarly qualitative studies, albeit taking into account the different research designs. It is not possible to provide estimates of the prevalence of particular view points in this study, however, and trends can only be compared in the most general ways with such studies. The recruitment of interview participants by means of an invitation extended to respondents in a random survey sample was both a strength and a limitation. Participants were chosen from those who agreed to be interviewed from the wider sample of those who responded to the survey. Aware that people on low incomes are less likely to respond to mail surveys we targeted a further 20 people in order to address this potential imbalance. Nevertheless people from culturally and linguistically diverse backgrounds were under-represented. While the gender distribution was roughly similar in each area, despite efforts to achieve representativeness, older people generally were more willing and more likely to be interviewed. This resulted in a sample favouring the older age groups, with 14 (18%) aged 18-35 years; 27 (34%) aged 36-55 years; and 39 (49%) aged over 56 years. Finally while four areas were chosen for their diversity, it is difficult to generalise to other areas given the influence of historical and other contextual factors.

## Conclusion

Notwithstanding the limited scope for comparison with other studies for reasons associated with variable research designs, the findings in this study are evocative of a kind of collective inertia within the public health field. The lack of congruence between explanations and public policy responses suggests that public health arguments directed at addressing the social determinants of health have not become absorbed into bodies of lay knowledge. Rather our findings suggest that lay theories relating to solutions to health inequities reflect a distinct strain of individualism. Tesh [28] noted some time ago that there are strong and usually hidden pressures towards individualism in public health policy and so it is not surprising that people reflect such responses.

The decision to extend beyond the question of explanation to theorizing solutions was an important methodological lever in this respect. As Martin [17] proposed, engaging with lay knowledge as opposed to mere lay beliefs has the potential to offer insights that may complement, challenge or hold a mirror to technical expertise in order to generate a different order of knowledge. In a tangible way this approach enabled us to integrate technical expertise and democratic participation, taking the contribution of lay knowledge 'up a notch', and simultaneously holding a mirror to the public health field.

That such a view reveals inconsistencies between explanation and solution need not be regarded as inevitable if the findings in other fields are any guide. For example, research indicates a remarkable consistency between the beliefs people hold about solutions to problems like obesity and depression and the bio-medical or psychological causes on which they are based [29]. The inconsistencies observed in this study suggest a specific failure to focus attention on comprehensible solutions in response to analysis of social/structural determinants, highlighting the dominance of individualism as the hallmark of neo-liberal public policies of the past two decades [30]. As such this finding reinforces the need identified by the Commission on the Social Determinants of Health to provide 'training on the social determinants of health to policy actors, stakeholders, and practitioners and invest[ment] in raising public awareness' [1]. Our study suggests that producing 'expert' knowledge on the causes of health inequalities will be insufficient to generate the public and political will for their implementation. In addition, increasing understanding of collective actions to address the structural causes is an important project for those with a desire to reduce health inequities.

## Competing interests

The authors declare that they have no competing interests.

## Authors' contributions

CP participated in the design of the study and in the collection and analysis of qualitative data, and prepared the preliminary draft of manuscript. FB conceived of the study, participated in its design and coordination and helped to draft the manuscript. AZ participated in the design of the study and in the collection and analysis of quantitative data and helped to draft the manuscript. All authors read and approved the final manuscript.

## Pre-publication history

The pre-publication history for this paper can be accessed here:

http://www.biomedcentral.com/1471-2458/11/67/prepub
